# Genetic Drivers of Ileal Neuroendocrine Tumors

**DOI:** 10.3390/cancers13205070

**Published:** 2021-10-10

**Authors:** Darren R. Carpizo, Chris R. Harris

**Affiliations:** Department of Surgery, University of Rochester Medical Center, 601 Elmwood Avenue, Rochester, NY 14642, USA; Darren_Carpizo@URMC.rochester.edu

**Keywords:** ileal, neuroendocrine

## Abstract

**Simple Summary:**

Although ileal neuroendocrine tumors are the most common tumors of the small intestine, they are not well-defined at the genetic level. Unlike most cancers, they have an unusually low number of mutations, and also lack recurrently mutated genes. Moreover ileal NETs have been difficult to study in the laboratory because there were no animal models and because cell lines were generally unavailable. But recent advances, including the first ileal NET mouse model as well as methods for culturing patient tumor samples, have been described and have already helped to identify IGF2 and CDK4 as two of the genetic drivers for this tumor type. These advances may help in the development of new treatments for patients.

**Abstract:**

The genetic causes of ileal neuroendocrine tumors (ileal NETs, or I-NETs) have been a mystery. For most types of tumors, key genes were revealed by large scale genomic sequencing that demonstrated recurrent mutations of specific oncogenes or tumor suppressors. In contrast, genomic sequencing of ileal NETs demonstrated a distinct lack of recurrently mutated genes, suggesting that the mechanisms that drive the formation of I-NETs may be quite different than the cell-intrinsic mutations that drive the formation of other tumor types. However, recent mouse studies have identified the IGF2 and RB1 pathways in the formation of ileal NETs, which is supported by the subsequent analysis of patient samples. Thus, ileal NETs no longer appear to be a cancer without genetic causes.

## 1. Introduction

Neuroendocrine tumors are the most common types of tumors within the small intestine, with an incidence of 1.05 per 100,000 in the United States [1,2]. Small intestinal NETs usually reside in the terminal ileum or cecum [3], where they are known as ileal NETs or I-NETs. I-NETs are slow growing tumors with low mitotic indices, and are often called “carcinoids” to distinguish them from faster growing carcinomas. I-NETs are difficult to detect early, and in 71% of initial diagnoses the tumor has already metastasized regionally or distally [4]. The average age at first diagnosis is 66 [4]. Survival is 98 months and 103 months for patients diagnosed with distant stage neuroendocrine tumors of the caecum or small intestine, respectively [1]. Carcinoids can also originate from other tissues such as lung, but the small intestine is the most common site of origin [5].

Although this review is focused on the genetic drivers of well-differentiated I-NETs, it should also be mentioned that the small intestine can develop two additional types of neuroendocrine tumors. Small intestinal neuroendocrine carcinomas (SI-NECs) are higher grade and less differentiated tumors than I-NETs, and they use different driver genes. SI-NECs are also much less common than I-NETs, and respond to different treatments [6,7]. Another type of NET, duodenal NETs (D-NETs), are found in the duodenum and are also less common than I-NETs. Duodenal and ileal NETs have different cells of origin and different driver genes, as shown by the fact that D-NETs may occur in patients with certain alleles of MEN1 and CDKN1B, whereas ileal NETs do not [8,9]. In certain cases, this review will reference studies of “small intestinal NETs,” which will indicate that datasets may have intermixed I-NETs with D-NETs and/or SI-NECs. The data from such studies may not be strictly applicable to well-differentiated ileal NETs.

**Figure 1:**
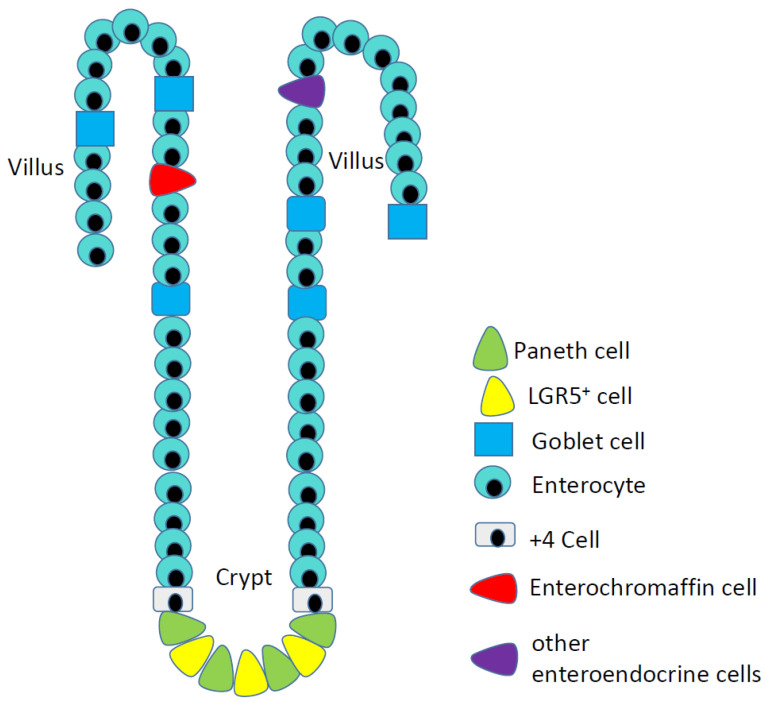
Epithelial cells of the ileum, including the rare serotonin-producing enterochromaffin cells (red cells) that are the origins of ileal neuroendocrine tumors.

I-NETs arise from serotonin-producing enterochromaffin (EC) cells of the ileum (Figure 1). These cells are quite rare: less than 1% of the epithelial cells of the ileum are enteroendocrine, while the serotonin-expressing EC cells are only a subset of the many types of enteroendocrine cells. I-NETs usually remain well-differentiated and indeed these tumors often secrete serotonin, such that patients can experience elevated serotonin levels along with symptoms related to serotonin vasoactivity. In some patients, high serotonin expression results in a condition known as carcinoid syndrome, which is characterized by facial flushing; abdominal cramps; diarrhea; hypotension; and fibrosis of the heart. Carcinoid syndrome is more common in women, and particularly common in patients with liver metastases [5]. Patients with primary I-NETs without associated liver metastases usually do not experience symptoms related to elevated serotonin levels due to the effect of first pass metabolism of the liver as serotonin metabolites by the liver are no longer vasoactive. Patients with I-NETs can excrete excessive amounts of a precursor of serotonin, 5-hydroxyindoleacetic acid, which can be detected using a simple urine test.

The liver is the most common site of the distant spread of I-NETs and as a result there are a variety of liver directed therapies that are often used to treat metastatic I-NETs. Surgical resection is considered to be the most effective treatment for I-NETs, however ablative and transarterial embolization therapies, such as bland and radioembolization, have shown a benefit [10,11].

Some of the more common types of cancer treatments are often not effective against ileal NETs. For instance, I-NETs generally do not respond to cytotoxic chemotherapies, and indeed current NCCN guidelines indicate that cytotoxic chemotherapies should only be considered for well-differentiated NETs if other options (e.g., surgery; somatostatin inhibitors; mTOR inhibitors) have been ineffective or are not feasible. There are also no precision medicine options for patients with I-NETs, due to a lack of recurrently mutated, actionable genes. Finally, I-NETs are also thought to be poor candidates for immunotherapies. In a phase II trial of PD1-targeting monoclonal antibody spartalizumab, patients with well-differentiated gastrointestinal NETs showed only a 3.1% overall response rate, which was below the trial’s pre-defined success criterion of 10% [12]. No partial responses to nivolumab/ipilimumab combination therapy were detected in five patients with low grade small intestinal NETs, while a partial response was detected in one patient with a high grade small intestinal neuroendocrine carcinoma (SI-NEC) [13]. The lack of response to immunotherapies may be due to the very low mutation rate within I-NETs, which results in a low number of tumor specific antigens.

I-NETs may grow faster after they metastasize to the liver. There are cases in which patients have very large liver metastases that express specific I-NET markers such as CDX2, yet the patients lack detectable primary tumors presumably because their primary I-NETs are so small. It is possible that the ileum expresses a growth factor that slows down the growth of I-NETs, or that the liver expresses a growth factor that accelerates the growth of I-NETs. For instance, somatostatin is an I-NET inhibitor (see next section) that is produced by enteroendocrine cells within the ileum but not by cells of the liver, whereas IGF2 is an activator of I-NET tumorigenesis (see below) that is usually produced at higher levels in the liver than in the small intestine.

## 2. A Major Role for Somatostatin in Ileal Net Biology

Somatostatin was isolated as a hormone that could prevent secretion of growth hormone [14], and was later shown also to decrease the secretion of serotonin and to relieve symptoms of carcinoid syndrome in patients with I-NETs [15]. Because somatostatin has a very short half-life, a longer-lived analogue known as octreotide was developed [16]. Although octreotide was approved in 1988 as a treatment to alleviate the symptoms of carcinoid syndrome, it was not until 2009 that octreotide was also shown to slow the growth of I-NETs [17]. Octreotide and somatostatin are bound by the somatostatin receptor SSTR2, which is highly expressed by ileal NETs as well as by NETs that develop in other organs. The molecular mechanism by which octreotide slows the growth of I-NETs is not established, but this drug affects the phosphatidylinositol 3-kinase pathway in pituitary neuroendocrine cells [18] and showed an effect on the TGF-β pathway in a pancreatic neuroendocrine cell line [19]. Interestingly, I-NETs do not have recurrent mutations in any of the presumed biochemical targets of somatostatin.

The somatostatin analogue DOTATATE has been linked to ^177^Lutetium, which upon decay produces a high energy beta electron that can damage DNA [20]. I-NET cells expressing SSTR2 take up ^177^Lu-DOTATATE, leading to DNA damage and cell death. This type of therapy is commonly referred to as peptide receptor radionuclide therapy (PRRT). NETTER-1 was a landmark clinical trial that led to the approval of PRRT for metastatic I-NETs. In NETTER-1, 229 patients with well differentiated metastatic midgut NETs were randomized to receive either ^177^Lu-DOTATATE plus long acting octreotide (30 mg, n = 116 patients), or long acting octreotide (60 mg, 113 patients). Progression free survival after 20 months was significantly longer in the PRRT + octreotide arm than in the octreotide alone arm (65.2% vs. 10.8%). At the 2018 ASCO meeting, a follow up was reported which showed that the median overall survival was 27.4 months in the octreotide arm and not reached in the PRRT + octreotide arm.

Somatostatin analogues have also improved the imaging of I-NETs. Early MRI or CT scans often failed to identify small I-NETs, but imaging of these smaller NETs was improved by use of single photon emission CT to detect ^111^Indium attached to octreotide analogue pentetreotide [21]. Newer, more sensitive nuclear imaging studies use positron emission with computed tomography (PET/CT) to detect radionuclides such as ^68^Ga-DOTATATE and less commonly ^18^F-DOPA or ^11^C-5-HTP. The use of phase contrast has also improved detection of small I-NETs by MRI and CT. 

## 3. The mTOR Pathway

The importance of the mTOR pathway in NET biology was first suggested by the appearance of pancreatic NETs in patients with tuberous sclerosis, which is caused by naturally occurring alleles of TSC1 or TSC2 that are known to activate mTOR [22,23,24,25,26]. mTOR inhibitors were also shown to slow growth of pancreatic NETs in RIP-TAG mice, a genetically engineered mouse model in which insulinomas arise due to expression of the oncogenic SV40 T-antigen under control of the insulin promoter [27]. Subsequently, patients with pancreatic NETs were treated with the mTOR inhibitor everolimus in a large clinical trial [28], in which patients with ileal NETs were also included. In a follow-up trial, the mTOR inhibitor everolimus was shown to increase progression-free survival in patients with advanced I-NETs [29]. The FDA approved everolimus as a treatment for advanced I-NETs in 2017. Everolimus also affects the growth of the I-NET cell line GOT1 [30]. The genetic mechanism by which the mTOR pathway is activated in I-NETs remains unclear. Ingenuity pathway analysis of copy number changes in I-NETs indicates that the mTOR pathway can be altered by rare, nonrecurrent, low copy number amplifications of AKT1, AKT2, mTOR, and PIK3CD [31]. Uncommon, nonrecurrent alterations of genes linked to the mTOR pathway were also detected using ingenuity pathway analysis of DNA methylation alterations in I-NETs [32].

## 4. I-Nets Lack Genes That Are Recurrently Mutated

It is notable that the importance of the SSTR2 and mTOR pathways in I-NET biology were chiefly discovered by clinicians, with little contribution from basic scientists and bioinformaticists. It has been difficult to study the basic science of I-NETs due to a lack of cell lines and animal models, while bioinformatics analyses have been confounded by a lack of recurrently mutated genes.

Whole exome sequencing revealed that ileal NETs have one of the lowest mutation rates of any tumor type [31,33]. The total number of mutations per I-NET is more similar to the frequency within pediatric tumors [33], in spite of the fact that the average age of diagnosis of ileal NETs is 66. There is also a distinct lack of recurrently mutated genes in I-NETs [31,33]. Whereas in other tumor types it is not uncommon to find genes that are mutated in 50–95% of cases, in I-NETs the gene that is most frequently associated with point mutations is CDKN1B, which is mutant in only 8% of cases [33,34,35,36]. This is a rather low mutation frequency for a tumor driver gene. Although tumor suppressor functions of CDKN1B are well known from mouse studies, it is notable that I-NETs have not been reported in CDKN1B mutant mice, nor have I-NETs been reported in patients with MEN4 syndrome, in which patients develop neuroendocrine tumors in multiple endocrine organs due to rare allelic forms of CDKN1B [8]. CDKN1B is a complex cancer gene because its protein, p27kip1, can act both as an activator or as a repressor of the RB1 tumor suppressor protein, by inhibiting CDK2 or by activating CDK4/Cyclin D, respectively [37,38]. In addition to CDKN1B point mutations, loss of copy of CDKN1B has been found in 1.5% to 14% of I-NET cases, depending on the study [33,34,35]. Loss of heterozygosity (LOH) is a characteristic of tumor suppressor genes but is rarely found in I-NETs with CDKN1B mutations, although it should be noted that CDKN1B appears to be a haploinsufficient tumor suppressor in other tumor types and in mouse studies [8,39].

Genes that are commonly mutated in other tumor types, such as p53, PIK3CA, or KRAS, are seldom mutated in I-NETs. In a recent targeted sequencing analysis of candidate tumor genes [36], fifty-seven cancer-related and neuroendocrine tumor-related genes were sequenced in 52 small intestinal NETs; copy number variations were also studied for another 40 candidate genes. Recurrently mutated genes included CDKN1B and APC (9.6% and 7.7% of cases, respectively) as well as CDKN2C (7.7%) and KRAS, PIK3CA, and TP53 (each altered in 3.8% of cases, or two patients each). Nonsynonymous APC mutations had previously been reported in five out of 30 ileal NETs [40]. APC is a key tumor suppressor of colorectal cancer, where its mutation often correlates with loss of heterozygosity and with nuclear localization of beta-catenin [41,42]. In three of the five I-NET patients with APC mutations, APC LOH and nuclear beta-catenin were also observed [40]. APC mutations would suggest that the WNT pathway is important for ileal NET biology. WNT pathway activation in I-NETs has also been suggested by the analysis of microRNAs that are differentially regulated in metastatic vs. primary I-NETs [43], and by the ingenuity pathway analysis of copy number variants in I-NETs [31].

However, it should be noted that APC mutations were less common in other studies of I-NETs [31,33,36]. Moreover, I-NETs have not been reported in mice with APC mutations [44], and I-NETs are extremely rare in patients with familial adenomatous polyposis [45], in whom alleles of APC lead to intestinal polyps and colorectal cancer. Hypermethylation of the CTNNB1 promoter, discussed below, also seems inconsistent with a role of the WNT pathway in ileal neuroendocrine tumorigenesis [46].

The low frequency of DNA mutations in I-NETs suggests that these tumors are not promoted by mutagens such as smoking or dietary carcinogens. In a comparison of the ratio of tumor mutational load relative to the estimated number of cell divisions by tumor precursor cells, organs with endocrine tumors (pancreatic islets, small intestine and duodenum) ranked as the lowest of all tumor types [47]. This analysis suggested that the mutations in endocrine tumors are strictly produced by replication errors and not by environmental mutagens. However, a recent study revealed that small intestinal NETs occurred at elevated rates in coal mining communities, suggesting a role for environmental factors [48].

## 5. Copy Number Variations

Although genes with recurrent point mutations are absent in I-NETs, some genes are recurrently altered by copy number alterations [31]. However, I-NETs lack the highly focal, high copy number amplifications of oncogenes that have identified tumor driver genes in other tumor types. Instead, recurrent copy number variants (CNVs) in I-NETs are generally due to gain or loss of a single copy of an entire chromosome. Common chromosomal gains in I-NETs include chromosomes 4, 5, 14, and 20, while common chromosome losses in I-NETs include chromosomes 9, 11, 16, and particularly 18 [49,50]. For most of these chromosomes, gains and losses occur in 10–30% of patient samples, depending on the percentage of metastatic samples in the data set. Chromosomal gains are more common in metastatic tumors.

Loss of an entire copy of chromosome 18 occurs in 40–75% of I-NETs depending on the study [51,52]. Chromosome 18 loss can be found in localized as well as in metastatic I-NETs, and may therefore be a marker of early disease. There is evidence for recurrent DNA methylation of LAMA3 and SERPINB5 genes on the remaining copy of chromosome 18, which suggests a “second hit” common to tumor suppressor genes [32,53]. LAMA3 is not a known tumor suppressor but SERPINB5, also known as maspin, has well-established tumor suppressor activity in breast cancer [54]. However, it is unclear whether neuroendocrine cells express maspin [55] and the ability of maspin to suppress I-NETs has not yet been rigorously tested using orthologous assays. SMAD2 and SMAD4 also reside on chromosome 18 and are potential tumor suppressors in the TGF-β pathway, which can crosstalk with the WNT pathway (see Section 4). No “second hits” of SMAD2 and SMAD4 have been reported in I-NETs and expression of these genes does not decrease at the mRNA level in I-NETs that have lost chromosome 18 [55]. Decreased SMAD4 expression was reported upon comparing metastatic with localized small intestinal NETs [56].

DCC is another chromosome 18 gene with well-established tumor suppressor activity in other tumor types [57]. DCC is best known as an axonal guidance molecule but it is also a dependence receptor that promotes cell death when its ligand, Netrin-1, is absent [58]. DCC expression is lowered in small intestinal NETs [55], consistent with a tumor suppressive role in these tumors. Lowered expression of another axonal guidance protein, Semaphorin 3, has also been reported in small intestinal NETs [59]. “Axonal guidance” was also detected by integrated pathway analysis of I-NET copy number alterations [31], and the neural morphology of EC cells certainly suggests that axonal guidance pathway components could play a role in I-NET biology.

Chromosome 20, which shows a copy number gain in some I-NETs, encodes the oncogene SRC. An increase in SRC copy number correlated with decreased patient survival in a small set of small intestinal NETs, and SRC mRNA was increased in samples with elevated SRC copy number [36]. The effect of SRC amplification on patient survival has not yet been repeated in a separate cohort. Perhaps more importantly, SRC activity is usually controlled post-translationally, whereas elevated SRC kinase activity has never been tested in I-NETs.

## 6. Mouse Modeling

The problem with modeling I-NETs in mice is two-fold. First, there is a lack of recurrently mutated genes, which are usually the starting point for designing a mouse model. Second, I-NETs are difficult to detect even in patients.

As mentioned above, CDKN1B is a known tumor suppressor gene that is mutated in a small percentage of patients with I-NETs. However, while CDKN1B mutant mice make a variety of tumors, I-NETs are not among the tumor types that have been reported in these animals [60]. It has been unclear whether this means that CDKN1B is not a suppressor of I-NETs or whether it is simply too difficult to detect I-NETs in mice.

For some purposes, xenograft models may adequately substitute for a lack of genetically engineered mouse models. However, a lack of available ileal NET cell lines has limited xenograft work for this tumor type. There is one human ileal NET cell line, GOT1, that has been reported to make xenografts [61], but for many years this cell line was not publicly available and as a result there has been very little work on GOT1-xenografted mice. It has also not been possible to generate patient-derived xenograft (PDX) models of I-NETs. Many researchers have implanted I-NETs into nude or NSG mice, but no PDX models have resulted. No I-NET PDXs are currently listed by the Human Cancer Models Initiative [62].

The first genetically engineered mouse model of ileal NETs was not reported until 2020 [63]. Ironically, these researchers were not trying to build an I-NET mouse model, but instead were studying pancreatic NETs in RIP-TAG (RT2) mice, which make insulinomas when they are in a C57Bl/6 (Black6, or B6) genetic background [64]. The researchers found that RT2 mice can make a different type of tumor, a nonfunctioning pancreatic NET (NF-PNET), when a RIP-TAG Black6 male was crossed with a wildtype female from the A/J genetic background [65,66]. This F1 hybrid is known as RT2AB6F1, because the mother’s genetic background (A) precedes the father’s background (B6). The reversed cross, in which RIP-TAG Black6 females are mated to A/J males, produce a related hybrid line known as RT2B6AF1. Interestingly, the latter animals developed not only NF-PNETs but also ileal NETs. Because I-NETs were found in RT2B6AF1 mice but not in RT2AB6F1 mice, it was thought that an imprinted gene might be required for the formation of I-NETs in mice. I-NET-related phenotypes in RT2B6AF1 mice were then shown to map at or near H19os, an imprinted gene that controls expression of insulin-like growth factor-2 (IGF2) [67,68]. Indeed, animals with I-NETs showed upregulation of both H19os and IGF2. The importance of IGF2 to I-NET development in mice was then established by demonstrating that a loss of copy of IGFBP1, a negative regulator of IGF2, enabled I-NETs to form in RT2B6 mice, which otherwise never develop I-NETs [63].

Importantly, IGF2 not only associated with I-NET formation in mice, but was also altered in a high percentage of patients. IGF2 is an imprinted oncogene, and loss of imprinting (LOI) of IGF2 was demonstrated in 57% of I-NET samples from patients [65]. Since IGF2 is upregulated by LOI in a high percentage of patients, and can also cause I-NETs to form in mice, IGF2 is the first gene that fits the strict criteria of a tumor driver gene in I-NETs.

RT2B6AF1 mice also provided clues about additional genes that are important in I-NET formation. Although I-NETs appeared in RT2B6AF1, these tumors were not detected in B6AF1 littermates, which do not encode SV40 T-antigen. SV40 T-antigen inactivates the powerful tumor suppressors RB1 and p53. In addition to being activated by CDKN2C and CDKN1B [38], which are mutated at low frequency in I-NETs, the RB1 tumor suppressor can also be activated by MIR1, which is produced by a gene on chromosome 18 and therefore shows a copy number loss in a majority of I-NETs [69]. MIR1 expression negatively correlated with CDK4 expression in I-NET patient samples, and MIR1 overexpression in NET cell lines caused G1 arrest, loss of expression of CDK4 and loss of phosphorylation of RB1. A number of studies have now detected downregulation of MIR1, and/or downregulation of its cistronic microRNA, MIR133, in metastatic I-NETs compared to primary I-NETs [63,70,71,72]. This suggests that advanced I-NETs are more likely to overexpress CDK4 and therefore that patients with advanced I-NETs may particularly benefit from CDK4/CDK6 inhibitor treatment. In the literature, only one patient with a small intestinal NET has been treated with a CDK4/CDK6 inhibitor. This patient was part of a basket trial of abemaciclib and showed a notable objective response to the drug [73]. Together, these data suggest that CDK4/CDK6 inhibitors should be considered for the treatment of patients with I-NETs that are otherwise not responding to standard of care.

p53, the other tumor suppressor inactivated by T-antigen in RT2B6AF1 mice, is almost never mutated in I-NETs, but overexpression of MDM2, the negative regulator of p53, has been noted in ileal NETs by our laboratory and also by Briest et al. [74]. Attenuation of p53 by MDM2 overexpression would help to explain the poor response of I-NETs to cytotoxic chemotherapy and might also be responsible for the elevated activity of mTOR.

An MDM2 inhibitor had a strong effect on the growth of GOT1, which is one of the only I-NET cell lines [75].

## 7. Epigenetic Alterations

Because of a lack of point mutations in I-NETs, epigenetic changes have long been suspected to play an important role in ileal NET biology. Although there are no recurrently mutated chromatin remodeling genes in I-NETs, both pancreatic NETs and lung NETs have mutations in these types of genes [76,77], suggesting that an altered chromatin structure is generally important in the biology of neuroendocrine tumors. In spite of this, studies of histone modifications are particularly lacking for I-NETs. In one study, the expression of the histone deacetylase HDAC5 was detected in I-NETs, and an HDAC inhibitor affected growth of the GOT1 cell line [50]. In a second study, H3K4 dimethylation was elevated in small intestinal NETs compared to hepatic cancers [78].

Most studies on epigenetic changes in I-NETs have focused on changes in DNA methylation. In the first of these studies, a high percentage (81%) of ileal NETs express the ORF1p protein of LINE-1 [79,80]. LINE-1 expression is a hallmark of cancer that indicates hypomethylated DNA [81]. A subsequent global DNA methylome analysis indicated that I-NETs indeed have genomic hypomethylation, although certain sites can also show hypermethylation [82,83]. By comparing I-NETs to normal adjacent tissue, a set of 21 genes was shown to distinguish metastatic small intestinal NETs from primary small intestinal NETs [32]. For these 21 genes, changes in DNA methylation correlated with changes in gene expression [82].

As described in the previous section, loss of imprinting of IGF2 is found in 57% of patients with I-NETs, and correlates with upregulated IGF2 expression in patient samples and in mice that develop I-NETs. LOI of IGF2 correlated with changes in DNA methylation in two imprinting control regions of the IGF2 promoter [63].

TCEB3C is a chromosome 18 gene with promoter hypermethylation and decreased expression in I-NETs, relative to normal adjacent tissue [84]. However, since TCEB3C is an imprinted gene, if its loss drives I-NET biology, then one might expect that all multifocal I-NETs (see next section) isolated from the same patient would lose the copy of chromosome 18 with imprinted TCEB3C. Instead, individual tumors from the same patient show no preference for which copy of chromosome 18 is lost [85].

A problem with comparing DNA methylation in I-NETs to DNA methylation in normal adjacent tissue is that the precursor cells of I-NETs are such a rare subpopulation within the normal adjacent tissue. A more technically challenging approach is to compare DNA methylation within tumors to DNA methylation within microdissected normal enterochromaffin cells. By microdissecting normal EC cells, Lloyd and colleagues demonstrated the hypermethylation of promoters of candidate genes, RASSF1A and CTNNB1 [46]. Hypermethylation of the same promoters in small intestinal NETs was also observed by another group [83]. Promoter hypermethylation of CTNNB1, a known oncogene, seems inconsistent with a role in tumorigenesis; however, RASSF1A is a tumor suppressor for which promoter hypermethylation has been detected in many cancers.

DNA methylation is seen as a key for early cancer detection. Cancer-specific and tissue-specific DNA methylation signatures in circulating DNA have been studied in order to develop multi-cancer screening tests, which will likely be offered as part of future patient wellness visits. However, even though ileal neuroendocrine tumors were included in a large search for cancer-specific DNA methylation markers, I-NETs are not listed among the 50 cancers that can be detected by the Galleri test that emerged from this study [86]. Indeed, Galleri advertises its ability to detect neuroendocrine tumors in the pancreas, appendix, large intestine and rectum, but not in the ileum or caecum. Thus, early detection will remain one of the biggest challenges for I-NETs, especially since most patients with this disease already have local or distant metastasis. Indeed, the problem of early detection even appears to be growing, as shown by an increase in the percentage of patients whose initial diagnosis is carcinoid syndrome, which is more common for metastatic disease [5]. Circulating RNAs and microRNAs that have been linked to small intestinal NETs may be able to aid in early detection [87,88].

## 8. Stromal Cell Involvement?

One of the singular properties of primary ileal NETs is their multifocal nature [89,90]. While few people will ever develop an I-NET within their lifetimes, patients who do develop this disease often have five or more tumors within their ileum. DNA analysis of individual tumors from patients with multiple lesions reveals that the multiple tumors arise independently of one another [85,91]. The low mutational load of I-NETs, combined with their multifocality, has suggested that drivers of I-NETs may be extrinsic, rather than intrinsic. Viruses and bacteria are cell-extrinsic causes of some cancers [92,93], and a virus has been linked to the multifocality of hepatocellular cancer [94]. However, no recurrent viral and bacterial sequences have yet to be associated with I-NETs, in spite of intensive research efforts.

Inflammation is a cell-extrinsic driver of some cancer types [95]. Indeed, there is an elevated incidence of I-NETs in patients with Crohn’s disease [96], which causes chronic inflammation of the ileum. However, not all patients with I-NETs have chronic inflammation, and chronic inflammation has not yet been linked to I-NET formation in mice. It is possible that I-NETs are detected in an elevated percentage of patients with Crohn’s disease simply because these patients are frequently screened for gastrointestinal problems, but it is also possible that the impact of inflammation has not been adequately studied for this disease.

As mentioned in a previous section, LOI of IGF2 can be found in 57% of I-NETs. Interestingly, LOI of IGF2 was also observed in matched normal tissue from these patients, including, in some cases, in normal tissue cut >20 cm from the tumor itself. Thus, a large zone of IGF2 overexpression can exist within the ilea of patients with I-NETs. Similarly large zones of IGF2 LOI have been reported in normal tissues from prostate cancer patients [97]. Since IGF2 is a driver of I-NETs, this zone of IGF2 overexpression may initiate multifocality. Notably, IGF2 may not have to be produced by the tumor cells themselves, but may instead be secreted by neighboring cells. Contributions by stromal cells would be consistent with the low mutation rate observed in I-NETs, and with the fact that LOI of IGF2 is observed in normal adjacent tissue. It would be interesting to engineer a mouse to overexpress IGF2 from some other intestinal cell type, such as a Paneth cell, to determine if gene expression from a neighboring cell type can lead to I-NET formation.

**Figure 2:**
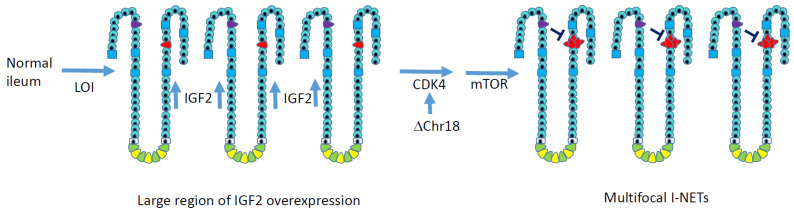
A working model for genetic events that lead to multifocal ileal NETs. Notably, these alterations are not caused by point mutations, which are rare in I-NETs. Overexpression of the growth factor IGF2 across a large region of the ileum occurs due to zonal loss of imprinting, which should increase the proliferation of enterochromaffin cells (red) in a number of intestinal crypts. Chromosome 18 loss decreases expression of resident gene MIR1-2, which normally suppresses CDK4 to maintain the RB1 tumor suppressor pathway. No recurrent genetic cause for mTOR activation has been elucidated in I-NETs, but mTOR inhibitors are clinically beneficial for I-NET patients. Even after tumorigenic events occur, the growth of primary ileal NETs often remains slow, possibly due to local production of the growth inhibitor somatostatin by neighboring enteroendocrine cells, which are shown in purple.

In Figure 2, we present a working model for ileal neuroendocrine tumorigenesis in which a zone of overexpression of IGF2 develops in the ileum, increasing the proliferation of enterochromaffin cells. A subsequent mitotic error results in the loss of one copy of chromosome 18 in multiple EC cells, which would decrease MIR1 expression, increase CDK4 expression, and attenuate the RB1 tumor suppressor pathway. Multiple tumors would then arise.

## 9. Epidemiological Studies

Since multifocality is also a property of familial cancers such as BRCA1 and BRCA2 cancers [98], it has been suggested that patients with multifocal I-NETs have inherited an allele that predisposes them to develop I-NETs. Indeed, families have been identified in which more than one member develops I-NETs [99,100,101,102,103,104]. In a heroic study, Wank and colleagues [101] discovered a large family that included two patients with clinical I-NETs. Many of the previously undiagnosed members of this family permitted the researchers to perform endoscopies, DOPA-PET, and even laproscopic surgeries to determine if other family members had previously undiagnosed I-NETs. DNA comparisons of the tumor-positive and tumor-negative family members allowed the mapping of a tumor-associating region on chromosome 10. Within this region, afflicted family members had a previously-unreported allelic variant of the gene for inositol polyphosphate multikinase (IPMK), from which four nucleotides were missing to cause a frame shift. The researchers also showed that this allele can reduce the activity of the p53 tumor suppressor protein. Notably, genetic alterations in IPMK have not been reported in spontaneous I-NETs, and rare alleles of IPMK are not reported in other families with multiple cases of I-NETs.

In another study, DNAs were collected from several small, unrelated families in which two or more members had developed I-NETs [99]. Whole exomic sequencing was performed on patients with inherited I-NETs and on their unaffected relatives, as well as patients with sporadic I-NETs and other case controls. The inherited cases in this study showed enrichment of a relatively rare though previously reported coding SNP in the MUTYH gene. MUTYH is a DNA repair gene, whose alteration would be expected to increase the number of tumor-specific mutations but the researchers did not indicate whether the mutation rate was lower in I-NETs lacking this MUTYH allele. This allele of MUTYH has also been associated with MUTYH loss of heterozygosity in certain pancreatic neuroendocrine tumors [105], indicating that the allele is a recessive tumor suppressor. No LOH of MUTYH was reported in the I-NET samples. Additional studies on SNP associations to I-NETs have focused on spontaneous and not familial I-NETs [106,107].

Tumor syndromes exist in which family members develop neuroendocrine tumors in many organs. Interestingly, patients with these “multiple endocrine neoplasia” or MEN syndromes never develop NETs in the ileum. For instance, members of families with MEN2 syndrome develop NETs in the adrenals, thyroid, and parathyroid, but not in the ileum [108], while members of families with MEN1 syndrome develop NETs in the pituitary, pancreas, parathyroid, and occasionally in the duodenum, lung, thymus, and stomach, but not in the ileum [9]. The fact that ileal NETs are not detected in MEN2 or MEN1 families strongly suggests that the genes that cause these syndromes, RET and MEN1 respectively, are not genetic drivers of I-NETs. Moreover, mutations in RET and MEN1 are not detected in I-NETs.

MEN4 syndrome is linked to allelic forms of CDKN1B, which is a gene that actually is altered in I-NETs. However, although members of MEN4 families develop NETs in the parathyroid, pituitary, and occasionally in the duodenum, no I-NETs have been reported [8]. Since very few cases of MEN4 have ever been studied, it is possible that in the future I-NETs may be reported in these families [8].

## 10. Transcriptomics

Comparisons of gene expression between I-NETs and their cells of origins have been complicated by the scarcity of normal EC cells, and thus have not been very helpful in demonstrating tumor driver genes or tumor driving pathways. On the other hand, comparisons between primary and metastasized tumors are less of a technical challenge, and have offered glimpses into potential metastatic driver pathways. In a recent study of primary and metastasized small intestinal NETs, there was an upregulation of several liver-specific genes and pathways in the metastases [109]. The upregulated pathways included complement and coagulation, and the upregulated genes included albumin and transferrin. Presumably the upregulation of liver genes was not due to contaminations of liver cells in the metastatic preparations, since other liver-specific genes were not detected. What is particularly interesting is that these same pathways and genes are upregulated in metastatic pancreatic neuroendocrine tumors [110], a tumor type that is presumed to have different tumor drivers than ileal NETs (e.g., MEN1 but not IGF2). Our laboratory also screened for pancreatic NET metastasis genes in mice and identified complement C5, then validated the importance of this gene both epistatically and pharmacologically [66]. From these data, I-NETs and pancreatic NETs may share common metastatic drivers.

## 11. In Vitro Modeling of I-Nets

A subset of the pathways that are probably important in I-NET biology is shown in Table 1. It is notable that for many of these pathways, there have been no in vitro experiments to test for potential mechanisms and their importance. This is due to a lack of availability of such tools as I-NET cell lines. 

Developing I-NET cell lines has been very difficult. Several cell lines, including KRJ-1, H-STS, and L-STS, were originally thought to be derived from I-NETs but were later shown to be lymphoblastoid cell lines [111]. The CNDT2.5 cell line was thought to derive from a tumor isolated from a patient with an I-NET, but later analysis revealed that the cell line was unrelated to that patient [112] and the source of CNDT2.5, along with its tumor of origin, remains a mystery. The Cancer Cell Line Factory reported a new cell line model of ileal neuroendocrine, CCLF_RCRF1049T, which was submitted to American Type Culture Collection in 2018 [113]. However, as of early 2021, this cell line was still not available from ATCC.

HC45 is an I-NET cell line that was produced by transfecting an ileal NET with SV40 T-antigen [114]. P-STS and GOT-1 are I-NET cell lines that have not been publicly available, presumably due to material transfer restrictions [115,116]. However, the GOT-1 cell line may now be available to the broader research community as our lab was recently granted an MTA for its use of GOT-1. This is a very favorable development for future ileal NET research.

A lack of cell lines has caused researchers to test ideas about I-NETs using cell lines derived from NETs of other organs, including NCI-H727, which was derived from a lung carcinoid; QGP1, which was derived from a pancreatic somatostatinoma [117]; and BON1, which was derived from a pancreatic carcinoid [118]. A mouse line, STC1, was also used, and was derived from a secretin-expressing neuroendocrine tumor of the duodenum [119]. Unfortunately, the genetics of I-NETs, which are not found in MEN1 families, are likely to differ substantially from the genetics of NETs from lung, pancreas, or duodenum, which are found in MEN1 families.

The struggle to generate cell lines also forced researchers into adopting other approaches. The generation of spheroids from I-NET patient samples was recently reported [120]. Organoids have been difficult to generate from I-NETs, but I-NET organoids were reported at recent research meetings and these studies are expected to be published within the next year.

## 12. Conclusions

**Figure 3:**
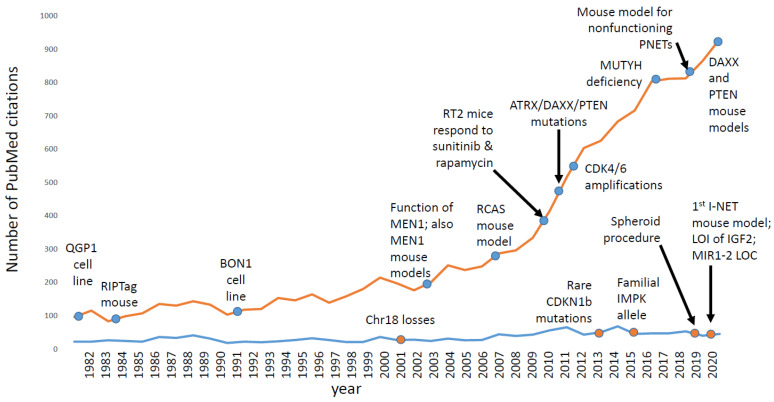
The number of pubmed references for the search terms “ileal + neuroendocrine + tumors” is shown by year using a blue line, and is compared to the pubmed references for the search terms “pancreatic + neuroendocrine + tumors“ (orange line). Ileal neuroendocrine tumors are actually more common in the clinic, but draw less research interest and less research funding due to a lack of cell line models, mouse models, and recurrently mutated genes. We speculate that recent discoveries for generating I-NET spheroids and mice, as well as new information about I-NET driver genes, will increase I-NET research interest in the future. Also noted are the years in which some of the key basic science results were published for each tumor type. The ileal neuroendocrine tumor references can be found in this paper; pancreatic neuroendocrine tumor references can be found in a separate review [125].

As shown in Figure 3, research interest in ileal NETs has not changed very much for many years. In contrast, research interest in pancreatic NETs (PNETs), which are less common than I-NETs in the clinic, has grown substantially over the same period of time. For PNETs, there are several mouse models and cell line models [64,65,121,122,123,124], which have helped basic scientists to ask scientific questions, to generate hypotheses, to test predictions, and to win grants. Indeed, over the years, the NIH has awarded several RO1 grants to studies with a PNET focus but none to a project with an I-NET focus. Scientists that study I-NETs have depended upon private foundations to fund their work, and often I-NETs are more of a side project than a lab’s main focus. Thus, one of the challenges of the field has been how to make I-NET research less of a side project and more of a focus.

Several recent advances in I-NET research should increase the appeal of researching this type of tumor. These advances include the identification of the first driver genes, development of the first mouse model, publication of a method for generating patient-derived spheroids, and possibly soon-to-be published methods for generating patient derived organoids. The availability of organoids and spheroids may finally allow the study of downstream targets of somatostatin and IGF2 in I-NETs as well as the importance of candidate tumor genes like MIR1-2, RASSF1A, APC, or CDKN1B. Indeed, the number of critical pathways shown in Table 1 will surely increase in the near future

In spite of a lack of knowledge about the genetic causes of this disease, treatment options for I-NETs have been increasing rapidly, including approvals for octreotide in 2013, for lanreotide in 2015, for everolimus in 2017, and for ^177^Lu-DOTATATE in 2018 [126]. While these clinical successes were accomplished with very little help from basic science, it seems possible that basic science will finally begin to contribute to the number of clinical options as well. For instance, studies of ileal NETs in RT2AB6F1 mice implicated IGF2, CDK4/6, and MDM2 in I-NET biology. IGF2-targeting and MDM2-targeting drugs have been in clinical trials for other tumor types, and CDK4/6 inhibitors are already approved for patients with breast cancer. Moreover, the ability to generate spheroids and organoids should allow large sets of candidate drug therapies to be tested in vitro, either for sets of I-NETs or even for individual patients, which may open personalized medicine as an option for patients with I-NETs despite a lack of tumor point mutations.

## Figures and Tables

**Table 1:** Some of the key pathways in Ileal NETs.

Pathway	Gene(s)	Clinical Evidence	Orthologous Evidence	Additional Notes	Key References
Somatostatin receptor-2	SSTR2	Somatostatin analogues approved for treatment of patients with I-NETs	GOT1 cell line responds to somatostatin analogues	SSTR2 is naturally expressed by EC cells.	[17,20,61]
mTOR	mTOR	mTOR inhibitors approved for treatment of patients with I-NETs	GOT1 cell line responds to everolimus	Nonrecurrent CNVs and promoter methylations in mTOR pathway	[20,30,31,32]
IGF2	IGF2	57% of I-NETs show loss of imprinting of IGF2	I-NETs form in RT2B6 and in RT2B6AF2 mice due to IGF2 pathway genes	Pathway downstream of IGF2 is unestablished in I-NETs	[63]
RB1	MIR1-2 CDKN1B CDKN2C	MIR1-2 shows loss of copy in 60% of I-NETs; CDKN1B is mutant in 8%; CDKN2C is mutant in 8%	I-NETs form in mice expressing SV40 T-antigen, which inactivates RB1		[33,34,35,36,69]
p53	MDM2 IPMK	MDM2 overproduction in patient samples; frame-shifted IPMK allele associates with familial I-NETs	I-NETs form in mice expressing SV40 T-antigen; MDM2 inhibitors prevent growth of GOT1	I-NETs often respond poorly to cytotoxic chemotherapies, suggestive of low p53 activity	[74,75,101]

## References

[1] Dasari A., Shen C., Halperin D., Zhao B., Zhou S., Xu Y., Shih T., Yao J.C. (2017). Trends in the Incidence, Prevalence, and Survival Outcomes in Patients With Neuroendocrine Tumors in the United States. JAMA Oncol..

[2] Scott A.T., Howe J.R. (2018). Management of Small Bowel Neuroendocrine Tumors. J. Oncol. Pract..

[3] Strosberg J. (2012). Neuroendocrine tumours of the small intestine. Best Pract. Res. Clin. Gastroenterol..

[4] Yao J.C., Hassan M., Phan A., Dagohoy C., Leary C., Mares J.E., Abdalla E.K., Fleming J.B., Vauthey J.N., Rashid A. (2008). One hundred years after “carcinoid”: Epidemiology of and prognostic factors for neuroendocrine tumors in 35,825 cases in the United States. J. Clin. Oncol..

[5] Halperin D.M., Shen C., Dasari A., Xu Y., Chu Y., Zhou S., Shih Y.T., Yao J.C. (2017). Frequency of carcinoid syndrome at neuroendocrine tumour diagnosis: A population-based study. Lancet Oncol..

[6] Rindi G., Wiedenmann B. (2020). Neuroendocrine neoplasia of the gastrointestinal tract revisited: Towards precision medicine. Nat. Rev. Endocrinol..

[7] Kawasaki K., Fujii M., Sato T. (2018). Gastroenteropancreatic neuroendocrine neoplasms: Genes, therapies and models. Dis. Model. Mech..

[8] Alrezk R., Hannah-Shmouni F., Stratakis C.A. (2017). MEN4 and CDKN1B mutations: The latest of the MEN syndromes. Endocr. Relat. Cancer.

[9] Kamilaris C.D.C., Stratakis C.A. (2019). Multiple Endocrine Neoplasia Type 1 (MEN1): An Update and the Significance of Early Genetic and Clinical Diagnosis. Front. Endocrinol. (Lausanne).

[10] Givi B., Pommier S.J., Thompson A.K., Diggs B.S., Pommier R.F. (2006). Operative resection of primary carcinoid neoplasms in patients with liver metastases yields significantly better survival. Surgery.

[11] Scott A.T., Breheny P.J., Keck K.J., Bellizzi A.M., Dillon J.S., O’Dorisio T.M., Howe J.R. (2019). Effective cytoreduction can be achieved in patients with numerous neuroendocrine tumor liver metastases (NETLMs). Surgery.

[12] Yao J.C., Strosberg J., Fazio N., Pavel M.E., Bergsland E., Ruszniewski P., Halperin D.M., Li D., Tafuto S., Raj N. (2021). Spartalizumab in metastatic, well/poorly-differentiated neuroendocrine neoplasms. Endocr. Relat. Cancer.

[13] Patel S.P., Othus M., Chae Y.K., Giles F.J., Hansel D.E., Singh P.P., Fontaine A., Shah M.H., Kasi A., Baghdadi T.A. (2020). A Phase II Basket Trial of Dual Anti-CTLA-4 and Anti-PD-1 Blockade in Rare Tumors (DART SWOG 1609) in Patients with Nonpancreatic Neuroendocrine Tumors. Clin. Cancer Res..

[14] Brazeau P., Vale W., Burgus R., Ling N., Butcher M., Rivier J., Guillemin R. (1973). Hypothalamic polypeptide that inhibits the secretion of immunoreactive pituitary growth hormone. Science.

[15] Frolich J.C., Bloomgarden Z.T., Oates J.A., McGuigan J.E., Rabinowitz D. (1978). The carcinoid flush. Provocation by pentagastrin and inhibition by somatostatin. N. Engl. J. Med..

[16] Bauer W., Briner U., Doepfner W., Haller R., Huguenin R., Marbach P., Petcher T.J., Pless J. (1982). SMS 201-995: A very potent and selective octapeptide analogue of somatostatin with prolonged action. Life Sci..

[17] Rinke A., Muller H.H., Schade-Brittinger C., Klose K.J., Barth P., Wied M., Mayer C., Aminossadati B., Pape U.F., Blaker M. (2009). Placebo-controlled, double-blind, prospective, randomized study on the effect of octreotide LAR in the control of tumor growth in patients with metastatic neuroendocrine midgut tumors: A report from the PROMID Study Group. J. Clin. Oncol..

[18] Theodoropoulou M., Zhang J., Laupheimer S., Paez-Pereda M., Erneux C., Florio T., Pagotto U., Stalla G.K. (2006). Octreotide, a somatostatin analogue, mediates its antiproliferative action in pituitary tumor cells by altering phosphatidylinositol 3-kinase signaling and inducing Zac1 expression. Cancer Res..

[19] Leu F.P., Nandi M., Niu C. (2008). The effect of transforming growth factor beta on human neuroendocrine tumor BON cell proliferation and differentiation is mediated through somatostatin signaling. Mol. Cancer Res..

[20] Strosberg J., El-Haddad G., Wolin E., Hendifar A., Yao J., Chasen B., Mittra E., Kunz P.L., Kulke M.H., Jacene H. (2017). Phase 3 Trial of (177)Lu-Dotatate for Midgut Neuroendocrine Tumors. N. Engl. J. Med..

[21] Krenning E.P., Kwekkeboom D.J., Bakker W.H., Breeman W.A., Kooij P.P., Oei H.Y., van Hagen M., Postema P.T., de Jong M., Reubi J.C. (1993). Somatostatin receptor scintigraphy with [111In-DTPA-D-Phe1]- and [123I-Tyr3]-octreotide: The Rotterdam experience with more than 1000 patients. Eur. J. Nucl. Med..

[22] Verhoef S., van Diemen-Steenvoorde R., Akkersdijk W.L., Bax N.M., Ariyurek Y., Hermans C.J., van Nieuwenhuizen O., Nikkels P.G., Lindhout D., Halley D.J. (1999). Malignant pancreatic tumour within the spectrum of tuberous sclerosis complex in childhood. Eur. J. Pediatr..

[23] Gutman A., Leffkowitz M. (1959). Tuberous sclerosis associated with spontaneous hypoglycaemia. Br. Med. J..

[24] Ilgren E.B., Westmoreland D. (1984). Tuberous sclerosis: Unusual associations in four cases. J. Clin. Pathol..

[25] Kim H., Kerr A., Morehouse H. (1995). The association between tuberous sclerosis and insulinoma. AJNR Am. J. Neuroradiol..

[26] Davoren P.M., Epstein M.T. (1992). Insulinoma complicating tuberous sclerosis. J. Neurol. Neurosurg. Psychiatry.

[27] Chiu C.W., Nozawa H., Hanahan D. (2010). Survival benefit with proapoptotic molecular and pathologic responses from dual targeting of mammalian target of rapamycin and epidermal growth factor receptor in a preclinical model of pancreatic neuroendocrine carcinogenesis. J. Clin. Oncol..

[28] Pavel M.E., Hainsworth J.D., Baudin E., Peeters M., Horsch D., Winkler R.E., Klimovsky J., Lebwohl D., Jehl V., Wolin E.M. (2011). Everolimus plus octreotide long-acting repeatable for the treatment of advanced neuroendocrine tumours associated with carcinoid syndrome (RADIANT-2): A randomised, placebo-controlled, phase 3 study. Lancet.

[29] Yao J.C., Fazio N., Singh S., Buzzoni R., Carnaghi C., Wolin E., Tomasek J., Raderer M., Lahner H., Voi M. (2016). Everolimus for the treatment of advanced, non-functional neuroendocrine tumours of the lung or gastrointestinal tract (RADIANT-4): A randomised, placebo-controlled, phase 3 study. Lancet.

[30] Zitzmann K., Ruden J., Brand S., Goke B., Lichtl J., Spottl G., Auernhammer C.J. (2010). Compensatory activation of Akt in response to mTOR and Raf inhibitors—A rationale for dual-targeted therapy approaches in neuroendocrine tumor disease. Cancer Lett..

[31] Banck M.S., Kanwar R., Kulkarni A.A., Boora G.K., Metge F., Kipp B.R., Zhang L., Thorland E.C., Minn K.T., Tentu R. (2013). The genomic landscape of small intestine neuroendocrine tumors. J. Clin. Investig..

[32] Karpathakis A., Dibra H., Pipinikas C., Feber A., Morris T., Francis J., Oukrif D., Mandair D., Pericleous M., Mohmaduvesh M. (2017). Progressive epigenetic dysregulation in neuroendocrine tumour liver metastases. Endocr. Relat. Cancer.

[33] Francis J.M., Kiezun A., Ramos A.H., Serra S., Pedamallu C.S., Qian Z.R., Banck M.S., Kanwar R., Kulkarni A.A., Karpathakis A. (2013). Somatic mutation of CDKN1B in small intestine neuroendocrine tumors. Nat. Genet..

[34] Maxwell J.E., Sherman S.K., Li G., Choi A.B., Bellizzi A.M., O’Dorisio T.M., Howe J.R. (2015). Somatic alterations of CDKN1B are associated with small bowel neuroendocrine tumors. Cancer Genet..

[35] Crona J., Gustavsson T., Norlen O., Edfeldt K., Akerstrom T., Westin G., Hellman P., Bjorklund P., Stalberg P. (2015). Somatic Mutations and Genetic Heterogeneity at the CDKN1B Locus in Small Intestinal Neuroendocrine Tumors. Ann. Surg. Oncol..

[36] Simbolo M., Vicentini C., Mafficini A., Fassan M., Pedron S., Corbo V., Mastracci L., Rusev B., Pedrazzani C., Landoni L. (2018). Mutational and copy number asset of primary sporadic neuroendocrine tumors of the small intestine. Virchows Arch..

[37] Larrea M.D., Liang J., Da Silva T., Hong F., Shao S.H., Han K., Dumont D., Slingerland J.M. (2008). Phosphorylation of p27Kip1 regulates assembly and activation of cyclin D1-Cdk4. Mol. Cell Biol..

[38] Alexander K., Hinds P.W. (2001). Requirement for p27(KIP1) in retinoblastoma protein-mediated senescence. Mol. Cell Biol..

[39] Le Toriellec E., Despouy G., Pierron G., Gaye N., Joiner M., Bellanger D., Vincent-Salomon A., Stern M.H. (2008). Haploinsufficiency of CDKN1B contributes to leukemogenesis in T-cell prolymphocytic leukemia. Blood.

[40] Bottarelli L., Azzoni C., Pizzi S., D’Adda T., Silini E.M., Bordi C., Rindi G. (2013). Adenomatous polyposis coli gene involvement in ileal enterochromaffin cell neuroendocrine neoplasms. Hum. Pathol..

[41] Morin P.J., Sparks A.B., Korinek V., Barker N., Clevers H., Vogelstein B., Kinzler K.W. (1997). Activation of beta-catenin-Tcf signaling in colon cancer by mutations in beta-catenin or APC. Science.

[42] Sparks A.B., Morin P.J., Vogelstein B., Kinzler K.W. (1998). Mutational analysis of the APC/beta-catenin/Tcf pathway in colorectal cancer. Cancer Res..

[43] Arvidsson Y., Rehammar A., Bergstrom A., Andersson E., Altiparmak G., Sward C., Wangberg B., Kristiansson E., Nilsson O. (2018). miRNA profiling of small intestinal neuroendocrine tumors defines novel molecular subtypes and identifies miR-375 as a biomarker of patient survival. Mod. Pathol..

[44] Moser A.R., Dove W.F., Roth K.A., Gordon J.I. (1992). The Min (multiple intestinal neoplasia) mutation: Its effect on gut epithelial cell differentiation and interaction with a modifier system. J. Cell Biol..

[45] July L.V., Northcott K.A., Yoshida E.M., Carr D.M., Owen D.A. (1999). Coexisting carcinoid tumors in familial adenomatous polyposis-associated upper intestinal adenomas. Am. J. Gastroenterol..

[46] Zhang H.Y., Rumilla K.M., Jin L., Nakamura N., Stilling G.A., Ruebel K.H., Hobday T.J., Erlichman C., Erickson L.A., Lloyd R.V. (2006). Association of DNA methylation and epigenetic inactivation of RASSF1A and beta-catenin with metastasis in small bowel carcinoid tumors. Endocrine.

[47] Tomasetti C., Vogelstein B. (2015). Cancer etiology. Variation in cancer risk among tissues can be explained by the number of stem cell divisions. Science.

[48] VanDerslice J., Taddie M.C., Curtin K., Miller C., Yu Z., Hemmert R., Cannon-Albright L.A., Neklason D.W. (2020). Early life exposures associated with risk of small intestinal neuroendocrine tumors. PLoS ONE.

[49] Andersson E., Sward C., Stenman G., Ahlman H., Nilsson O. (2009). High-resolution genomic profiling reveals gain of chromosome 14 as a predictor of poor outcome in ileal carcinoids. Endocr. Relat. Cancer.

[50] Andersson E., Arvidsson Y., Sward C., Hofving T., Wangberg B., Kristiansson E., Nilsson O. (2016). Expression profiling of small intestinal neuroendocrine tumors identifies subgroups with clinical relevance, prognostic markers and therapeutic targets. Mod. Pathol..

[51] Lollgen R.M., Hessman O., Szabo E., Westin G., Akerstrom G. (2001). Chromosome 18 deletions are common events in classical midgut carcinoid tumors. Int. J. Cancer.

[52] Wang G.G., Yao J.C., Worah S., White J.A., Luna R., Wu T.T., Hamilton S.R., Rashid A. (2005). Comparison of genetic alterations in neuroendocrine tumors: Frequent loss of chromosome 18 in ileal carcinoid tumors. Mod. Pathol..

[53] Verdugo A.D., Crona J., Starker L., Stalberg P., Akerstrom G., Westin G., Hellman P., Bjorklund P. (2014). Global DNA methylation patterns through an array-based approach in small intestinal neuroendocrine tumors. Endocr. Relat. Cancer.

[54] Streuli C.H. (2002). Maspin is a tumour suppressor that inhibits breast cancer tumour metastasis in vivo. Breast Cancer Res..

[55] Nieser M., Henopp T., Brix J., Stoss L., Sitek B., Naboulsi W., Anlauf M., Schlitter A.M., Kloppel G., Gress T. (2017). Loss of Chromosome 18 in Neuroendocrine Tumors of the Small Intestine: The Enigma Remains. Neuroendocrinology.

[56] Hofving T., Elias E., Rehammar A., Inge L., Altiparmak G., Persson M., Kristiansson E., Johansson M.E., Nilsson O., Arvidsson Y. (2021). SMAD4 haploinsufficiency in small intestinal neuroendocrine tumors. BMC Cancer.

[57] Fearon E.R., Cho K.R., Nigro J.M., Kern S.E., Simons J.W., Ruppert J.M., Hamilton S.R., Preisinger A.C., Thomas G., Kinzler K.W. (1990). Identification of a chromosome 18q gene that is altered in colorectal cancers. Science.

[58] Boussouar A., Tortereau A., Manceau A., Paradisi A., Gadot N., Vial J., Neves D., Larue L., Battistella M., Leboeuf C. (2020). Netrin-1 and Its Receptor DCC Are Causally Implicated in Melanoma Progression. Cancer Res..

[59] Bollard J., Massoma P., Vercherat C., Blanc M., Lepinasse F., Gadot N., Couderc C., Poncet G., Walter T., Joly M.O. (2015). The axon guidance molecule semaphorin 3F is a negative regulator of tumor progression and proliferation in ileal neuroendocrine tumors. Oncotarget.

[60] Fero M.L., Randel E., Gurley K.E., Roberts J.M., Kemp C.J. (1998). The murine gene p27Kip1 is haplo-insufficient for tumour suppression. Nature.

[61] Nilsson O., Kolby L., Bernhardt P., Forssell-Aronsson E., Johanson V., Ahlman H. (2004). GOT1 xenografted to nude mice: A unique model for in vivo studies on SSTR-mediated radiation therapy of carcinoid tumors. Ann. N. Y. Acad. Sci..

[62] hcmi-searchable-catalog.nci.nih.gov.

[63] Contractor T., Clausen R., Harris G., Rosenfeld J., Carpizo D., Tang L., Harris C. (2020). IGF2 drives formation of ileal neuroendocrine tumors in patients and mice. Endocr. Relat. Cancer.

[64] Hanahan D. (1985). Heritable formation of pancreatic beta-cell tumours in transgenic mice expressing recombinant insulin/simian virus 40 oncogenes. Nature.

[65] Kobayashi S., Contractor T., Vosburgh E., Du Y.N., Tang L.H., Clausen R., Harris C.R. (2019). Alleles of Insm1 determine whether RIP1-Tag2 mice produce insulinomas or nonfunctioning pancreatic neuroendocrine tumors. Oncogenesis.

[66] Contractor T., Kobayashi S., da Silva E., Clausen R., Chan C., Vosburgh E., Tang L.H., Levine A.J., Harris C.R. (2016). Sexual dimorphism of liver metastasis by murine pancreatic neuroendocrine tumors is affected by expression of complement C5. Oncotarget.

[67] Berteaux N., Aptel N., Cathala G., Genton C., Coll J., Daccache A., Spruyt N., Hondermarck H., Dugimont T., Curgy J.J. (2008). A novel H19 antisense RNA overexpressed in breast cancer contributes to paternal IGF2 expression. Mol. Cell Biol..

[68] Tran V.G., Court F., Duputie A., Antoine E., Aptel N., Milligan L., Carbonell F., Lelay-Taha M.N., Piette J., Weber M. (2012). H19 antisense RNA can up-regulate Igf2 transcription by activation of a novel promoter in mouse myoblasts. PLoS ONE.

[69] Contractor T., Harris C.R. (2020). Loss of copy of MIR1-2 increases CDK4 expression in ileal neuroendocrine tumors. Oncogenesis.

[70] Li S.C., Essaghir A., Martijn C., Lloyd R.V., Demoulin J.B., Oberg K., Giandomenico V. (2013). Global microRNA profiling of well-differentiated small intestinal neuroendocrine tumors. Mod. Pathol..

[71] Ruebel K., Leontovich A.A., Stilling G.A., Zhang S., Righi A., Jin L., Lloyd R.V. (2010). MicroRNA expression in ileal carcinoid tumors: Downregulation of microRNA-133a with tumor progression. Mod. Pathol..

[72] Miller H.C., Frampton A.E., Malczewska A., Ottaviani S., Stronach E.A., Flora R., Kaemmerer D., Schwach G., Pfragner R., Faiz O. (2016). MicroRNAs associated with small bowel neuroendocrine tumours and their metastases. Endocr. Relat. Cancer.

[73] Fujiwara Y., Tamura K., Kondo S., Tanabe Y., Iwasa S., Shimomura A., Kitano S., Ogasawara K., Turner P.K., Mori J. (2016). Phase 1 study of abemaciclib, an inhibitor of CDK 4 and 6, as a single agent for Japanese patients with advanced cancer. Cancer Chemother. Pharmacol..

[74] Briest F., Grass I., Sedding D., Mobs M., Christen F., Benecke J., Fuchs K., Mende S., Kaemmerer D., Sanger J. (2018). Mechanisms of Targeting the MDM2-p53-FOXM1 Axis in Well-Differentiated Intestinal Neuroendocrine Tumors. Neuroendocrinology.

[75] Reuther C., Heinzle V., Nolting S., Herterich S., Hahner S., Halilovic E., Jeay S., Wuerthner J.U., Aristizabal Prada E.T., Spottl G. (2018). The HDM2 (MDM2) Inhibitor NVP-CGM097 Inhibits Tumor Cell Proliferation and Shows Additive Effects with 5-Fluorouracil on the p53-p21-Rb-E2F1 Cascade in the p53wild type Neuroendocrine Tumor Cell Line GOT1. Neuroendocrinology.

[76] Fernandez-Cuesta L., Peifer M., Lu X., Sun R., Ozretic L., Seidal D., Zander T., Leenders F., George J., Muller C. (2014). Frequent mutations in chromatin-remodelling genes in pulmonary carcinoids. Nat. Commun..

[77] Jiao Y., Shi C., Edil B.H., de Wilde R.F., Klimstra D.S., Maitra A., Schulick R.D., Tang L.H., Wolfgang C.L., Choti M.A. (2011). DAXX/ATRX, MEN1, and mTOR pathway genes are frequently altered in pancreatic neuroendocrine tumors. Science.

[78] Magerl C., Ellinger J., Braunschweig T., Kremmer E., Koch L.K., Holler T., Buttner R., Luscher B., Gutgemann I. (2010). H3K4 dimethylation in hepatocellular carcinoma is rare compared with other hepatobiliary and gastrointestinal carcinomas and correlates with expression of the methylase Ash2 and the demethylase LSD1. Hum. Pathol..

[79] Choi I.S., Estecio M.R., Nagano Y., Kim D.H., White J.A., Yao J.C., Issa J.P., Rashid A. (2007). Hypomethylation of LINE-1 and Alu in well-differentiated neuroendocrine tumors (pancreatic endocrine tumors and carcinoid tumors). Mod. Pathol..

[80] Harris C.R., Normart R., Yang Q., Stevenson E., Haffty B.G., Ganesan S., Cordon-Cardo C., Levine A.J., Tang L.H. (2010). Association of nuclear localization of a long interspersed nuclear element-1 protein in breast tumors with poor prognostic outcomes. Genes Cancer.

[81] Rodic N., Sharma R., Sharma R., Zampella J., Dai L., Taylor M.S., Hruban R.H., Iacobuzio-Donahue C.A., Maitra A., Torbenson M.S. (2014). Long interspersed element-1 protein expression is a hallmark of many human cancers. Am. J. Pathol..

[82] Karpathakis A., Dibra H., Pipinikas C., Feber A., Morris T., Francis J., Oukrif D., Mandair D., Pericleous M., Mohmaduvesh M. (2016). Prognostic Impact of Novel Molecular Subtypes of Small Intestinal Neuroendocrine Tumor. Clin. Cancer Res..

[83] Fotouhi O., Adel Fahmideh M., Kjellman M., Sulaiman L., Hoog A., Zedenius J., Hashemi J., Larsson C. (2014). Global hypomethylation and promoter methylation in small intestinal neuroendocrine tumors: An in vivo and in vitro study. Epigenetics.

[84] Edfeldt K., Ahmad T., Akerstrom G., Janson E.T., Hellman P., Stalberg P., Bjorklund P., Westin G. (2014). TCEB3C a putative tumor suppressor gene of small intestinal neuroendocrine tumors. Endocr. Relat. Cancer.

[85] Zhang Z., Makinen N., Kasai Y., Kim G.E., Diosdado B., Nakakura E., Meyerson M. (2020). Patterns of chromosome 18 loss of heterozygosity in multifocal ileal neuroendocrine tumors. Genes Chromosomes Cancer.

[86] Liu M.C., Oxnard G.R., Klein E.A., Swanton C., Seiden M.V., Consortium C. (2020). Sensitive and specific multi-cancer detection and localization using methylation signatures in cell-free DNA. Ann. Oncol..

[87] Oberg K., Modlin I.M., De Herder W., Pavel M., Klimstra D., Frilling A., Metz D.C., Heaney A., Kwekkeboom D., Strosberg J. (2015). Consensus on biomarkers for neuroendocrine tumour disease. Lancet Oncol..

[88] Malczewska A., Frampton A.E., Mato Prado M., Ameri S., Dabrowska A.F., Zagorac S., Clift A.K., Kos-Kudla B., Faiz O., Stebbing J. (2019). Circulating MicroRNAs in Small-bowel Neuroendocrine Tumors: A Potential Tool for Diagnosis and Assessment of Effectiveness of Surgical Resection. Ann. Surg..

[89] Gangi A., Siegel E., Barmparas G., Lo S., Jamil L.H., Hendifar A., Nissen N.N., Wolin E.M., Amersi F. (2018). Multifocality in Small Bowel Neuroendocrine Tumors. J. Gastrointest. Surg..

[90] Yantiss R.K., Odze R.D., Farraye F.A., Rosenberg A.E. (2003). Solitary versus multiple carcinoid tumors of the ileum: A clinical and pathologic review of 68 cases. Am. J. Surg. Pathol..

[91] Katona T.M., Jones T.D., Wang M., Abdul-Karim F.W., Cummings O.W., Cheng L. (2006). Molecular evidence for independent origin of multifocal neuroendocrine tumors of the enteropancreatic axis. Cancer Res..

[92] Kostic A.D., Gevers D., Pedamallu C.S., Michaud M., Duke F., Earl A.M., Ojesina A.I., Jung J., Bass A.J., Tabernero J. (2012). Genomic analysis identifies association of Fusobacterium with colorectal carcinoma. Genome Res..

[93] Cover T.L., Blaser M.J. (2009). Helicobacter pylori in health and disease. Gastroenterology.

[94] Miao R., Luo H., Zhou H., Li G., Bu D., Yang X., Zhao X., Zhang H., Liu S., Zhong Y. (2014). Identification of prognostic biomarkers in hepatitis B virus-related hepatocellular carcinoma and stratification by integrative multi-omics analysis. J. Hepatol..

[95] Greten F.R., Grivennikov S.I. (2019). Inflammation and Cancer: Triggers, Mechanisms, and Consequences. Immunity.

[96] West N.E., Wise P.E., Herline A.J., Muldoon R.L., Chopp W.V., Schwartz D.A. (2007). Carcinoid tumors are 15 times more common in patients with Crohn’s disease. Inflamm. Bowel. Dis..

[97] Bhusari S., Yang B., Kueck J., Huang W., Jarrard D.F. (2011). Insulin-like growth factor-2 (IGF2) loss of imprinting marks a field defect within human prostates containing cancer. Prostate.

[98] Bergthorsson J.T., Ejlertsen B., Olsen J.H., Borg A., Nielsen K.V., Barkardottir R.B., Klausen S., Mouridsen H.T., Winther K., Fenger K. (2001). BRCA1 and BRCA2 mutation status and cancer family history of Danish women affected with multifocal or bilateral breast cancer at a young age. J. Med. Genet..

[99] Dumanski J.P., Rasi C., Bjorklund P., Davies H., Ali A.S., Gronberg M., Welin S., Sorbye H., Gronbaek H., Cunningham J.L. (2017). A MUTYH germline mutation is associated with small intestinal neuroendocrine tumors. Endocr. Relat. Cancer.

[100] Moertel C.G., Dockerty M.B. (1973). Familial occurrence of metastasizing carcinoid tumors. Ann. Intern. Med..

[101] Sei Y., Zhao X., Forbes J., Szymczak S., Li Q., Trivedi A., Voellinger M., Joy G., Feng J., Whatley M. (2015). A Hereditary Form of Small Intestinal Carcinoid Associated With a Germline Mutation in Inositol Polyphosphate Multikinase. Gastroenterology.

[102] Wale R.J., Williams J.A., Beeley A.H., Hughes E.S. (1983). Familial occurrence in carcinoid tumours. Aust. N. Z. J. Surg..

[103] Kinova S., Duris I., Kovacova E., Stvrtina S., Galbavy S., Makaiova I. (2001). Malignant carcinoid in two brothers. Bratisl. Lek. Listy.

[104] Pal T., Liede A., Mitchell M., Calender A., Narod S.A. (2001). Intestinal carcinoid tumours in a father and daughter. Can. J. Gastroenterol..

[105] Scarpa A., Chang D.K., Nones K., Corbo V., Patch A.M., Bailey P., Lawlor R.T., Johns A.L., Miller D.K., Mafficini A. (2017). Whole-genome landscape of pancreatic neuroendocrine tumours. Nature.

[106] Du Y., Ter-Minassian M., Brais L., Brooks N., Waldron A., Chan J.A., Lin X., Kraft P., Christiani D.C., Kulke M.H. (2016). Genetic associations with neuroendocrine tumor risk: Results from a genome-wide association study. Endocr. Relat. Cancer.

[107] Walsh K.M., Choi M., Oberg K., Kulke M.H., Yao J.C., Wu C., Jurkiewicz M., Hsu L.I., Hooshmand S.M., Hassan M. (2011). A pilot genome-wide association study shows genomic variants enriched in the non-tumor cells of patients with well-differentiated neuroendocrine tumors of the ileum. Endocr. Relat. Cancer.

[108] Wells S.A., Pacini F., Robinson B.G., Santoro M. (2013). Multiple endocrine neoplasia type 2 and familial medullary thyroid carcinoma: An update. J. Clin. Endocrinol. Metab..

[109] Wang Q., Yu C. (2020). Expression profiling of small intestinal neuroendocrine tumors identified pathways and gene networks linked to tumorigenesis and metastasis. Bioscience Rep..

[110] Chan C.S., Laddha S.V., Lewis P.W., Koletsky M.S., Robzyk K., Da Silva E., Torres P.J., Untch B.R., Li J., Bose P. (2018). ATRX, DAXX or MEN1 mutant pancreatic neuroendocrine tumors are a distinct alpha-cell signature subgroup. Nat. Commun..

[111] Hofving T., Arvidsson Y., Almobarak B., Inge L., Pfragner R., Persson M., Stenman G., Kristiansson E., Johanson V., Nilsson O. (2018). The neuroendocrine phenotype, genomic profile and therapeutic sensitivity of GEPNET cell lines. Endocr. Relat. Cancer.

[112] Ellis L.M., Samuel S., Sceusi E. (2010). Varying opinions on the authenticity of a human midgut carcinoid cell line--letter. Clin. Cancer Res..

[113] Cancer Cell Line Factory. https://cellfactory.broadinstitute.org/#/verified-models.

[114] Stilling G.A., Zhang H., Ruebel K.H., Leontovich A.A., Jin L., Tanizaki Y., Zhang S., Erickson L.A., Hobday T., Lloyd R.V. (2007). Characterization of the functional and growth properties of cell lines established from ileal and rectal carcinoid tumors. Endocr. Pathol..

[115] Kolby L., Bernhardt P., Ahlman H., Wangberg B., Johanson V., Wigander A., Forssell-Aronsson E., Karlsson S., Ahren B., Stenman G. (2001). A transplantable human carcinoid as model for somatostatin receptor-mediated and amine transporter-mediated radionuclide uptake. Am. J. Pathol..

[116] Pfragner R., Behmel A., Hoger H., Beham A., Ingolic E., Stelzer I., Svejda B., Moser V.A., Obenauf A.C., Siegl V. (2009). Establishment and characterization of three novel cell lines—P-STS, L-STS, H-STS—derived from a human metastatic midgut carcinoid. Anticancer Res..

[117] Kaku M., Nishiyama T., Yagawa K., Abe M. (1980). Establishment of a carcinoembryonic antigen-producing cell line from human pancreatic carcinoma. Gan.

[118] Evers B.M., Townsend C.M., Upp J.R., Allen E., Hurlbut S.C., Kim S.W., Rajaraman S., Singh P., Reubi J.C., Thompson J.C. (1991). Establishment and characterization of a human carcinoid in nude mice and effect of various agents on tumor growth. Gastroenterology.

[119] Rindi G., Grant S.G., Yiangou Y., Ghatei M.A., Bloom S.R., Bautch V.L., Solcia E., Polak J.M. (1990). Development of neuroendocrine tumors in the gastrointestinal tract of transgenic mice. Heterogeneity of hormone expression. Am. J. Pathol..

[120] Ear P.H., Li G., Wu M., Abusada E., Bellizzi A.M., Howe J.R. (2019). Establishment and Characterization of Small Bowel Neuroendocrine Tumor Spheroids. J. Vis. Exp..

[121] Crabtree J.S., Scacheri P.C., Ward J.M., Garrett-Beal L., Emmert-Buck M.R., Edgemon K.A., Lorang D., Libutti S.K., Chandrasekharappa S.C., Marx S.J. (2001). A mouse model of multiple endocrine neoplasia, type 1, develops multiple endocrine tumors. Proc. Natl. Acad. Sci. USA.

[122] Biondi C.A., Gartside M.G., Waring P., Loffler K.A., Stark M.S., Magnuson M.A., Kay G.F., Hayward N.K. (2004). Conditional inactivation of the MEN1 gene leads to pancreatic and pituitary tumorigenesis but does not affect normal development of these tissues. Mol. Cell Biol..

[123] Shen H.C., He M., Powell A., Adem A., Lorang D., Heller C., Grover A.C., Ylaya K., Hewitt S.M., Marx S.J. (2009). Recapitulation of pancreatic neuroendocrine tumors in human multiple endocrine neoplasia type I syndrome via Pdx1-directed inactivation of Men1. Cancer Res..

[124] Wong C., Tang L.H., Davidson C., Vosburgh E., Chen W., Foran D.J., Notterman D.A., Levine A.J., Xu E.Y. (2019). Two well-differentiated pancreatic neuroendocrine tumor mouse models. Cell Death Differ..

[125] Mafficini A., Scarpa A. (2019). Genetics and Epigenetics of Gastroenteropancreatic Neuroendocrine Neoplasms. Endocr. Rev..

[126] Tsoli M., Chatzellis E., Koumarianou A., Kolomodi D., Kaltsas G. (2019). Current best practice in the management of neuroendocrine tumors. Ther. Adv. Endocrinol. Metab..

